# Qiviut cortisol is associated with metrics of health and other intrinsic and extrinsic factors in wild muskoxen (*Ovibos moschatus*)

**DOI:** 10.1093/conphys/coab103

**Published:** 2022-01-21

**Authors:** Juliette Di Francesco, Grace P S Kwong, Rob Deardon, Sylvia L Checkley, Gabriela F Mastromonaco, Fabien Mavrot, Lisa-Marie Leclerc, Susan Kutz

**Affiliations:** 1 Department of Ecosystem and Public Health, Faculty of Veterinary Medicine, University of Calgary, 3280 Hospital Drive NW, Calgary, Alberta T2N 4Z6, Canada; 2 Department of Population Health and Reproduction, School of Veterinary Medicine, University of California Davis, Davis, CA 95616, USA; 3 Faculty of Veterinary Medicine, University of Calgary, 3280 Hospital Drive NW, Calgary, Alberta T2N 4Z6, Canada; 4 Department of Community Health Sciences, Cumming School of Medicine, University of Calgary, 3280 Hospital Drive NW, Calgary, Alberta T2N 4Z6, Canada; 5 Department of Production Animal Health, Faculty of Veterinary Medicine, University of Calgary, 3280 Hospital Drive NW, Calgary, Alberta T2N 4Z6, Canada; 6 Department of Mathematics and Statistics, Faculty of Science, University of Calgary, 2500 University Drive NW, Calgary, Alberta T2N 1N4, Canada; 7 Reproductive Physiology Unit, Toronto Zoo, 361A Old Finch Avenue, Scarborough, Ontario M1B 5K7, Canada; 8 Department of Environment, Government of Nunavut, P.O. Box 377, Kugluktuk, Nunavut X0B 0E0, Canada

**Keywords:** Arctic, hair cortisol, hunter-based sampling, muskox, qiviut, wildlife

## Abstract

Glucocorticoid (GC) levels are increasingly and widely used as biomarkers of hypothalamic–pituitary–adrenal (HPA) axis activity to study the effects of environmental changes and other perturbations on wildlife individuals and populations. However, identifying the intrinsic and extrinsic factors that influence GC levels is a key step in endocrinology studies to ensure accurate interpretation of GC responses. In muskoxen, qiviut (fine woolly undercoat hair) cortisol concentration is an integrative biomarker of HPA axis activity over the course of the hair’s growth. We gathered data from 219 wild muskoxen harvested in the Canadian Arctic between October 2015 and May 2019. We examined the relationship between qiviut cortisol and various intrinsic (sex, age, body condition and incisor breakage) and extrinsic biotic factors (lungworm and gastrointestinal parasite infections and exposure to bacteria), as well as broader non-specific landscape and temporal features (geographical location, season and year). A Bayesian approach, which allows for the joint estimation of missing values in the data and model parameters estimates, was applied for the statistical analyses. The main findings include the following: (i) higher qiviut cortisol levels in males than in females; (ii) inter-annual variations; (iii) higher qiviut cortisol levels in a declining population compared to a stable population; (iv) a negative association between qiviut cortisol and marrow fat percentage; (v) a relationship between qiviut cortisol and the infection intensity of the lungworm *Umingmakstrongylus pallikuukensis*, which varied depending on the geographical location; and (vi) no association between qiviut cortisol and other pathogen exposure/infection intensity metrics. This study confirmed and further identified important sources of variability in qiviut cortisol levels, while providing important insights on the relationship between GC levels and pathogen exposure/infection intensity. Results support the use of qiviut cortisol as a tool to monitor temporal changes in HPA axis activity at a population level and to inform management and conservation actions.

## Teaser

Muskoxen are increasingly exposed to stressors in the rapidly changing Arctic. Hair cortisol, an indicator of stress, varied among years, sex, and geographical location in wild muskoxen and was associated with poorer body condition and lungworms. These results support use of hair cortisol as a tool for monitoring muskox health and informing management.

## Introduction

Climate warming and other anthropogenic changes are occurring at an unprecedented pace worldwide. Understanding the effects that these changes and their associated stressors have on wildlife species is essential for biodiversity conservation (IPCC, 2014; Ribera d’Alcalà, 2019). This is particularly relevant in high-latitude environments such as the Arctic, where climate changes have been especially rapid and pronounced and have multiple impacts, including an increased frequency of extreme weather events, the loss of sea ice, higher surface temperatures and modifications in species associations and abundance (AMAP, 2017; Cuyler *et al.*, 2020b).

Muskoxen (*Ovibos moschatus*), an iconic Arctic ungulate species, are essential for the nutrition, economy and culture of local Indigenous communities (Tomaselli *et al.*, 2018a; Cuyler *et al.*, 2020a). Over the past two decades, muskoxen have undergone substantial population declines on Banks and Victoria Islands in the western Canadian Arctic Archipelago (Tomaselli *et al.*, 2018b; Cuyler *et al.*, 2020b). These declines are linked to multiple factors, including icing events and large-scale disease-associated mortality and morbidity (Nagy and Gunn, 2009; Nagy *et al.,* 2009; Kutz *et al.,* 2017; Cuyler *et al.*, 2020b). Recent emerging pathogens and disease syndromes of concern include *Erysipelothrix rhusiopathiae*, brucellosis, orf (Parapoxvirus) and expanding lungworm infections (Kutz *et al.,* 2015; Tomaselli *et al.,* 2016, 2018b, 2019; Kafle *et al.,* 2020; Mavrot *et al.,* 2020). The recent population declines, along with emerging diseases and the very low genetic diversity of muskoxen (Prewer *et al.,* 2020), suggest that this species may be particularly threatened by the multiple environmental changes and associated stressors to which it is increasingly exposed (Kutz *et al.,* 2017; Cuyler *et al.*, 2020b).

The activation of the hypothalamic–pituitary–adrenal (HPA) axis, which leads to the release of glucocorticoids (GCs) (mainly cortisol in muskoxen; Koren *et al.,* 2012), is an important part of the physiological stress response in mammals and plays a key role in energy regulation (Romero and Butler, 2007; Busch and Hayward, 2009). GC levels are increasingly and widely used as biomarkers of HPA axis activity to study the effects of various environmental changes and challenges on wildlife individuals and populations (Busch and Hayward, 2009; Dantzer *et al.,* 2014). These hormones are incorporated and can be readily quantified in various biological matrices including blood, saliva, faeces and urine (in the form of metabolites), and hair (reviewed in Dantzer *et al.,* 2014). Among these matrices, blood, saliva, urine and faeces provide short-term information on an animal’s GC levels at a single point in time or over hours to days, whereas hair is thought to provide a cumulative measurement of GC levels during the period of the hair’s growth—this may be weeks to months depending on turnover patterns and sampling regime (Sheriff *et al.,* 2011; Russell *et al.,* 2012). Hair GC concentrations may, therefore, represent a non-specific integrative measure of all the stressors experienced by the animal over the course of the hair’s growth (Cattet *et al.,* 2014).

**Figure 1:**
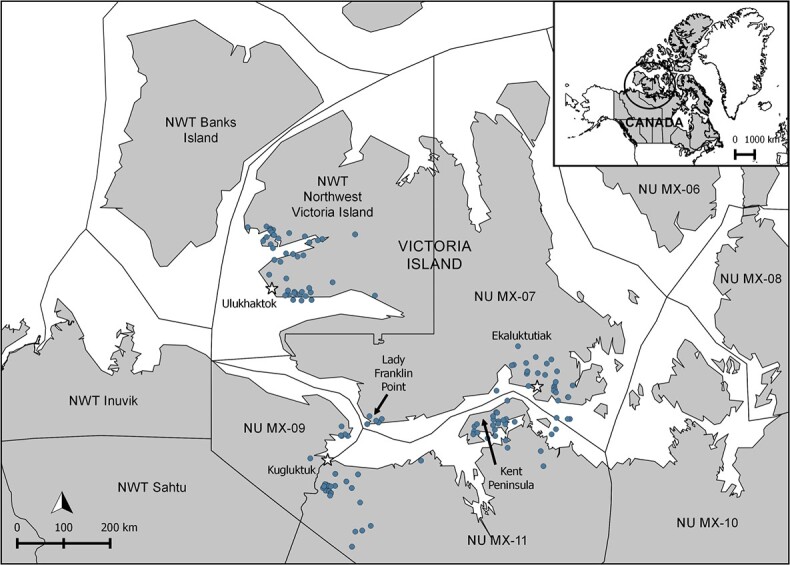
Map showing the muskox management units (MMUs) in the NWT and NU and the five specific geographical locations from which samples were obtained [communities of Ulukhaktok, Kugluktuk and Ekaluktutiak (black and white stars), and Lady Franklin Point and Kent Peninsula (black arrows)], with the geo-referenced harvesting locations of the muskoxen when available (blue points). Geographic coordinates were unavailable for 15 of the 211 muskoxen; these animals were assigned to a specific geographical location based on the MMUs in which they were harvested and on the community from which the kit was submitted (Ekaluktutiak, *n* = 8; Kugluktuk, *n* = 2; Ulukhaktok, *n* = 3; Kent Peninsula, *n* = 2). Map generated in QGIS version 2.8.9 using the shapefile from Cuyler *et al.*, 2020b. For the purposes of the analyses, animals from Ekaluktutiak, Lady Franklin Point and Ulukhaktok were grouped within the broad geographical location of Victoria Island, which is only divided into two MMUs because of political boundaries. Animals harvested near Kugluktuk (NU MX-09 and west part of NU MX-11) and on the Kent Peninsula (east part of NU MX-11) were treated as two separate broad geographical locations (i.e. west and east mainland, respectively), because of ecological and muskox health status differences (Leclerc, 2014; Walker *et al.,* 2005).

Hair GCs have been widely used to investigate the stress response of wildlife species to anthropogenic disturbances, extreme weather events and pathogens (e.g. Bryan *et al.,* 2015; Ewacha *et al.,* 2017; Fardi *et al.,* 2018; Madslien *et al.,* 2020). Less frequently, hair GCs have also been used to examine the possible consequences of elevated GCs on fitness indicators (e.g. survival probability, neonate birthweight and pregnancy success; Rakotoniaina *et al.,* 2017; Downs *et al.,* 2018). Even more rarely, studies have attempted to demonstrate the link between a stressor, elevated GCs and the effect of these hormones on fitness (but see Dulude-de Broin *et al.,* 2020). Hair GCs are influenced by a wide variety of extrinsic (e.g. season, social status) and intrinsic (e.g. sex, reproductive status) factors that may affect both baseline GC levels and the magnitude of the response to stressors, as well as the incorporation of GCs into the hair (Dantzer *et al.,* 2014; Heimbürge *et al.,* 2019). These predictable influencing factors must be identified and controlled for in each species of interest in order to adequately interpret GC responses to environmental changes and disturbances. While many studies have assessed the relationship between GC levels and a single or few extrinsic and/or intrinsic factors (e.g. Salas *et al.,* 2016; Fardi *et al.,* 2018), the simultaneous inclusion of multiple factors in the analyses is rare (but see Santos *et al.,* 2018). In particular, studies that investigated the association between GC levels and pathogens were generally focused on a single type of pathogen/parasite while only accounting for a few other extrinsic and/or intrinsic factors (e.g. Chapman *et al.,* 2006; Madslien *et al.,* 2020, but see Hoby *et al.,* 2006; Cizauskas *et al.,* 2015).

Cortisol concentration in the qiviut (fine woolly undercoat hair) of muskoxen can be used as a biomarker of HPA axis activity during the period of the hair’s growth (Di Francesco *et al.,* 2021). Here, we use a large-scale cross-sectional study of wild muskoxen in the Canadian Arctic to examine the relationship between qiviut cortisol levels and various intrinsic (sex, age, body condition and incisor breakage) and extrinsic biotic factors [lungworm and gastrointestinal (GI) parasite infection intensities, parasite richness, exposure to bacteria], as well as broader landscape and temporal features (geographical location, season and year). Building on our previous work (Di Francesco *et al.,* 2017), this study confirms and further identifies important sources of variability in qiviut cortisol levels.

## Material and methods

### Animals and sampling procedure

This study was approved by the Veterinary Sciences Animal Care Committee, University of Calgary (protocol #AC13-0121). Samples were obtained under the Wildlife Research Permit #2016-058 for Nunavut (NU) and the Wildlife Research Permits #WL500257, WL5004469 and WL500664 for the Northwest Territories (NWT).

Samples from 219 muskoxen, harvested as part of a community-based muskox health surveillance program in Ekaluktutiak and Kugluktuk, NU, and in Ulukhaktok, NWT, were obtained between October 2015 and May 2019 (Fig. 1). The surveillance program was developed through a partnership among these communities, Hunters and Trappers Organizations/Committees, guided hunting organizations, government biologists and academic researchers. Samples were obtained through individual subsistence, community and guided hunts.

**Figure 2:**
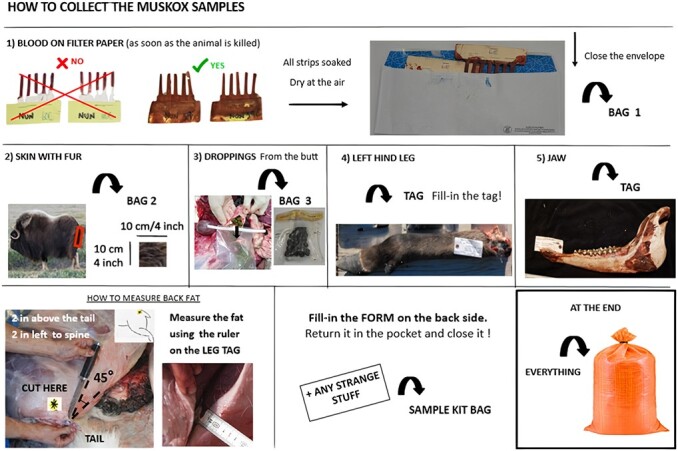
Instructions provided to the hunters with the kits to collect the samples.

Hunters and guides collected samples using standardized kits available to them at the local wildlife office and/or Hunters and Trappers Organization/Committee. Participation in this program was voluntary and they received financial compensation for each completed kit. Kits were modified from those described by Kutz *et al.* (2013b) for caribou. A fully completed kit would include the following: blood collected on Nobuto filter paper strips (Toyo Roshi Kaisha, Ltd, Tokyo, Japan), faeces, the lower left hind leg, the lower jaw and a piece of skin with fur from the rump measuring ~10 × 10 cm (Fig. 2). The geographic coordinates of the kill location, the age class and sex of the muskox, a measure of its back fat thickness, a subjective assessment of its body condition (i.e. categories ‘skinny’, ‘not bad’, ‘fat’ or ‘really fat’) and observations of abnormalities seen during butchering were recorded by the hunter using the datasheet provided with the kit (Supplementary Fig. 1). All samples were stored at approximately −20°C until analysis. Not all returned kits and information forms were complete, and the amount of a sample collected was sometimes not sufficient for all the tests to be performed (e.g. faecal samples and parasitology testing), which led to missing values in the dataset (Table 1; Supplementary Fig. 2).

**Table 1:** Variables, and their respective abbreviations, descriptions and sample sizes, evaluated as potential predictors of qiviut cortisol levels in the 211 muskoxen. None of the continuous variables were normally distributed, so all are summarized as median (range). Missing values (i.e. insufficient sample for all laboratory analyses or information not recorded on data sheet), except those for back fat thickness and incisor breakage score, were estimated through the Bayesian analyses

Information	Variable	Variable description	Number of muskoxen (%)	
General	Season (*season*)	*Categorical* Fall–early winter Mid-winter–late winter	75 136	(35.5) (64.5)
	Year (i.e. hair growth year) (*year*)	*Categorical* 2015 2016 2017 2018	28 41 80 62	(13.3) (19.4) (37.9) (29.4)
	Sex (*sex*)	*Categorical* Female Male	91 120	(43.1) (56.9)
	Age (*age*)	*Categorical* Adult Juvenile	191 20	(90.5) (9.5)
	Specific geographical location	*Categorical* Ekaluktutiak Kent Peninsula Kugluktuk Lady Franklin Point Ulukhaktok	39 30 70 13 59	(18.5) (14.2) (33.2) (6.2) (27.9)
	Broad geographical location (*location*)	*Categorical* East mainland (Kent Peninsula) Victoria Island (Ekaluktutiak, Lady Franklin Point and Ulukhaktok) West mainland (Kugluktuk)	30 111 70	(14.2) (52.6) (33.2)
Lungworms	*U. pallikuukensis* larval counts (*Up_lpg*)	*Continuous* 43.1 lpg (0–1669.8) Missing	200 11	(94.8) (5.2)
	*V. eleguneniensis* larval counts (*Ve_lpg*)	*Continuous* 1.3 lpg (0–442.7) Missing	200 11	(94.8) (5.2)
	Lungworm richness (*lung_richness*)	*Categorical* 0 (no lungworms detected) 1 (*Up* or *Ve* detected) 2 (both *Up* and *Ve* detected) Missing	28 54 118 11	(13.3) (25.6) (55.9) (5.2)
GI parasites	*Moniezia* spp. infection (*moniezia_YN*)	*Categorical* No Yes Missing	152 23 36	(72.0) (10.9) (17.1)
	*Eimeria* spp. oocyst counts (*eimeria_epg*)	*Continuous* 2.5 epg (0–822.6) Missing	175 36	(82.9) (17.1)
	Nematodirines egg counts (*nematodirines_epg*)	*Continuous* 0 epg (0–16.1) Missing	175 36	(82.9) (17.1)
	*M. marshalli* egg counts (*marshallagia_epg*)	*Continuous* 0 epg (0–10.4) Missing	175 36	(82.9) (17.1)
	GI parasite richness (*GI_richness*)	*Categorical* 0 (no GI parasites detected) 1 (1 GI parasite species/group detected) 2 (2 GI parasite species/groups detected) 3 (3 GI parasite species/groups detected) 4 (4 GI parasite species/groups detected) Missing	5 61 58 41 10 36	(2.4) (28.9) (27.5) (19.4) (4.7) (17.1)
Body condition	Metatarsus percentage marrow fat (*marrow_fat*)	*Continuous* 91.1% (9.9–94.8)^a^ Missing	197 14	(93.4) (6.6)
	Hunter condition assessment (*condition_hunter*)	*Categorical* Skinny Not bad Fat Really fat Missing	21 53 93 20 24	(9.9) (25.1) (44.1) (9.5) (11.4)
	Back fat thickness (*back_fat*)	*Continuous* 2 cm (0–6.4) Missing^b^	145 66	(68.7) (31.3)
Bacteria exposure	*E. rhusiopathiae* percentage positivity (*erysipelothrix_PP*)	*Continuous* 22.4% (1–496.1)^a^ Missing	203 8	(96.2) (3.8)
	*Brucella* serology (*brucella_serology*)	*Categorical* Negative Positive Missing	192 11 8	(91.0) (5.2) (3.8)
Jaw health	Incisor breakage score (*incisor_breakage*)	*Continuous* 0 (0–1) Missing^b^	152 59	(72.0) (28.0)
Hormone levels (outcome)	Qiviut cortisol levels (*qiviut_cortisol*)	*Continuous* 16.3 (3.8–84.5)		

^a^These values were divided by 100 for the statistical analyses.

^b^Not estimated through Bayesian analyses.

Samples were classified to two different seasons based on their collection date: late fall–early winter (last week of September to end of December) and mid-winter–late winter (January to mid-May). There was a large overlap between the growth periods of some of the late fall-early winter and mid-winter–late winter qiviut samples, as qiviut grows approximately from early April to late November with no growth observed between mid-December and March (Flood *et al.,* 1989; Robertson, 2000). However, this categorization was decided for consistency with a previous seasonal classification (Di Francesco *et al.,* 2017) and due to the high seasonality of some of the other health indicators, such as body condition. The year was defined as the year of qiviut growth, not the year of sample collection (i.e. muskoxen harvested in January through mid-May were assigned to the year prior to harvest because the qiviut sampled at the time of harvest had grown during the previous summer and fall).

When left blank or indicated as ‘unknown’ on the information sheet, the sex of the muskox was determined using genetic analyses as previously detailed by Di Francesco *et al.* (2017). The age was categorized as ‘juvenile’ (calves and yearlings) or ‘adult’ (>2 years) for the analyses. This was estimated either based on the teeth eruption patterns when the lower jaw was collected (Henrichsen and Grue, 1980) or by the hunter if the jaw was not available. If neither of these sources of information were available, the age class was determined based on the metatarsus length for muskoxen harvested on Victoria Island. We plotted the distribution of metatarsus length for animals of known age class and developed a cut-off of 16 cm, above which muskoxen were classified as adults and below which the age remained ‘unknown’ (Supplementary Fig. 3). The eight muskoxen with an ‘unknown’ age were removed from the statistical analyses, as we chose not to impute the missing data for this variable, leaving a total of 211 animals to be included.

### Sample analyses

#### Parasitology

Approximately 5 g from each faecal sample was weighed (as little as 2 g were used if the amount was limited) and analysed using the modified beaker Baermann technique for the presence of protostrongylid lungworm first stage larvae (L1) (Forrester and Lankester, 1997). Modifications were as follows: (i) tissue paper was replaced by a single layer of cheesecloth; (ii) samples sat for 5 min after removal of the faecal envelope before the supernatant was aspirated (using gentle vacuum suction) down to 15 ml and transferred in a test tube; (iii) the beaker was rinsed with 2 ml of tap water, which were added to the test tube; and (iv) samples were then centrifuged, supernatant aspirated down to 2 ml and, depending on the amount of L1 expected, either the entire sample or three 50 or 100 μl aliquots were analysed. The L1 recovered were identified microscopically under 40× magnification as *Umingmakstrongylus pallikuukensis* (*Up*) or *Varestrongylus eleguneniensis* (*Ve*) based on their caudal morphology following the keys developed by Kafle *et al.* (2015). Each species was quantified separately and L1 counts are expressed as larvae per gramme of faeces (lpg).

Approximately 2–4 g (depending on the amount available) from each faecal sample was weighed and evaluated for the presence of GI parasite eggs and oocysts using the modified Wisconsin double-centrifugation sugar flotation technique (Egwang and Slocombe, 1982). Modifications included straining the water-faecal mixture through a single layer of cheesecloth instead of a tea strainer and centrifuging the samples for 10 min at 1500 rpm both times. The eggs and oocysts recovered were counted and identified microscopically under 100× magnification to the group or species level (nematodirines (*Nematodirus* spp. and *Nematodirella* spp.), *Marshallagia marshallagi*, *Eimeria* spp. or *Moniezia* spp.) based on their morphological characteristics. The eggs of *Teladorsagia boreoarcticus* and *Ostertagia gruehneri*, two major GI parasites of muskoxen, were not counted as egg production occurs mainly throughout the summer (reviewed in Kutz *et al.,* 2012) and freezing of faecal samples results in a rapid decrease in the number of detectable eggs of these species (De Bruyn, 2010). All egg and oocyst counts are expressed as eggs per gramme of faeces (epg), except for *Moniezia* spp. for which only the absence or presence of eggs was recorded as entire proglottids are typically shed in the faeces and quantification of eggs in faeces is not considered representative of actual parasite burden (Taylor *et al.,* 2015).

We assumed that larval and egg/oocyst counts served as proxies for their corresponding parasite infection intensities (with the exception of *Moniezia* spp. as explained above). The correlations between the number of adult worms in the lungs or GI tract and the amount of L1, eggs or oocysts shed in the faeces have not been investigated in muskoxen. However, a study in Svalbard reindeer (*Rangifer tarandus platyrhynchus*) showed that faecal egg counts were positively associated with adult worm burden for *M. marshalli* (Irvine *et al.,* 2001). An experimental study on a limited number of muskoxen suggested a similar relationship between faecal larval counts and adult worm burden for *Up* (Kutz *et al.,* 1999).

Parasite richness was recorded for lungworms and GI parasites by counting the number of different species (lungworms) and species or groups (GI parasites) infecting each muskox.

#### Hormone analyses

For each animal, qiviut, intermediate hairs and guard hairs from an area free from obvious blood or other contamination were cut away from the skin using a scalpel blade. Qiviut was manually separated from guard and intermediate hairs using forceps and was divided into two subsamples of 0.05 g, which were independently analysed. Qiviut cortisol analyses were done at the Endocrinology Laboratory of the Toronto Zoo following the procedure detailed in Di Francesco *et al.* (2021). The mean concentration between the two subsamples, measured in nanograms of cortisol per gramme of qiviut (ng/g), was used as the final response variable. Duplicate subsamples had a median coefficient of variation [CV, calculated as (standard deviation/mean) × 100] of 12.70% (range = 0.14–83.26%).

#### Serology

Filter paper sets were removed from the freezer and air-dried at room temperature for at least 24 h. They were then eluted following the method described by Curry *et al.* (2011) and eluates were stored at −20°C until testing.

Eluates were tested for antibodies against *E. rhusiopathiae* with a modified enzyme-linked immunosorbent assay (ELISA) (Mavrot *et al.,* 2020). Results were standardized across all ELISA plates and expressed as percentage positivity (PP) of a reference muskox positive control. The PP was calculated as (OD_sample_ − OD_blank_)/(OD_cont_ − OD_blank_) × 100, with OD_sample_ corresponding to the optic density (OD) value measured for the sample, OD_cont_ to the OD value of the positive control, and OD_blank_ to the OD value of the ELISA plate’s blank well (Mavrot *et al.,* 2020).

Samples were tested for antibodies against *Brucella* using two different indirect enzyme-linked immunoassays (i-ELISA). All samples from hair growth year 2015 (*n* = 28) were analysed at the Arctic University of Norway, Research Group for Arctic Infection Biology (Tromsø, Norway; Nymo *et al.,* 2013). The remaining samples (*n* = 183) were analysed at the Canadian Food Inspection Agency National Brucellosis Reference Laboratory (Ottawa, Canada; Nielsen *et al.,* 1994, 2004). In both i-ELISAs, the antigen was smooth lipopolysaccharide from *Brucella abortus* and the enzyme conjugate was a peroxidase conjugated chimeric protein A/G (Nielsen *et al.,* 2004; Nymo *et al.,* 2013). Because of the clear cut-off between positive and negative samples, results were expressed as a positive/negative status.

#### Marrow fat measurement and other indices of body condition

The percentage of metatarsus marrow fat was measured following the protocol described by the CircumArctic Rangifer Monitoring and Assessment (CARMA) Network (2008), except marrow samples were dried at room temperature instead of in an oven (CARMA, 2008). The percentage of fat was calculated as (dry weight/fresh weight) × 100. We used the percentage of metatarsus marrow fat as a metric of body condition, along with the back fat thickness and the subjective body condition assessment by the hunter.

#### Lower jaw analysis

Lower jaws were assessed for incisor breakage (both permanent and deciduous). The incisor breakage score was calculated as number of incisors broken/number of incisors examined.

### Statistical analyses

The effects of the different intrinsic (sex, age, body condition, jaw health), extrinsic abiotic (season, hair growth year, broad geographical location) and extrinsic biotic (lungworms, GI parasites, bacteria exposure) factors (Table 1) on qiviut cortisol were assessed using a Bayesian approach. This method allows for the joint estimation of missing data values and model parameters and consequently takes into account the uncertainty due to missing data when estimating parameters (see Erler *et al.,* 2016). *Back_fat* and *incisor_breakage* were not included in the original modelling procedure as >25% of records for these variables were missing. The effects of these two variables and their corresponding interactions on qiviut cortisol were later assessed using the subset of animals for which they had been recorded by adding them into the final model.

Correlation analyses were performed to check for multicollinearity between the explanatory variables. This was done using Spearman’s rank correlation coefficient (r_s_) as these variables were not normally distributed (McCrum-Gardner, 2008). Biologically and ecologically plausible two-way interactions were originally identified through discussions among co-authors and based on the current knowledge in the available scientific literature (e.g. Baker *et al.,* 2013). Only those for which the data were fairly balanced across the various levels of the variables were considered. Tested interactions are presented in Table 2. To determine which interactions would be important to keep for model building, we fitted a model with all the main effects, to which we added independently each identified interaction to check the impact on the deviance information criterion (DIC) (Spiegelhalter *et al.,* 2002; Gelman *et al.,* 2004) and whether it had an ‘important’ effect on qiviut cortisol [based on the 95% credible interval (CrI)]. For the purpose of parsimony, we only retained the three interactions in the model that produced the best improvement (i.e. largest reduction) of the DIC when compared with the model including only all the main effects (i.e. *age* and *Ve_lpg*, *sex* and *year, year* and *erysipelothrix_PP*), as well as some others that did not substantially improve the DIC, but for which there was suggestive evidence that they had an ‘important’ effect (i.e. *location* and *Up_lpg*, *sex* and *season*; see Supplementary Table 1). The resulting model, which we term the ‘stage 2’ model, included the five interaction terms and all the main effects, except for *brucella_serology* because of its absence of effect, low prevalence and unbalanced data (e.g. no positive muskoxen in the west mainland). From that ‘stage 2’ model, we proceeded through manual backward elimination to simplify the model and sequentially removed the variables or group of variables (i.e. GI parasite egg/oocyst counts) that did not appear to have an ‘important’ effect on qiviut cortisol when considering their 95% CrI. With each removal, the impact on the DIC (Supplementary Table 2) and on the parameter posterior estimates of the other variables was checked. As indicated in Spiegelhalter *et al.* (2002), a difference in DIC of <2 was considered ‘not important’, with variables being removed when their elimination resulted in such an increase. When checking the impact of each variable’s removal on the parameter estimates of the other variables, we observed that the parameter estimates remained relatively stable across all models. An interaction was never included without its main effects also being included in the model.

**Table 2:** Biologically and ecologically plausible two-way interactions tested

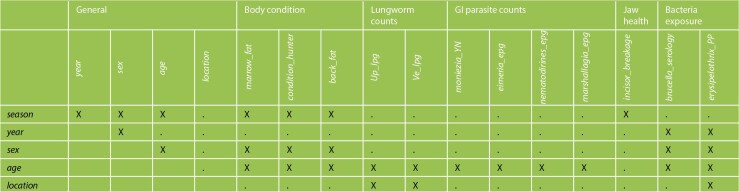

We considered our missing data as missing at random and consequently the missing data mechanism as ‘ignorable’ (i.e. the value of the variable is independent of the probability of it being missing) (Ma and Chen, 2018). For all model parameters, we assumed a relatively flat normal prior distribution with mean zero and standard deviation of 1000, so that the parameter estimates were unlikely to be influenced by our choice of prior distribution. Additional prior information was included for the random imputation of certain variables: (i) specific geographical location for disease exposure; (ii) specific geographical location and age for lungworm larval counts; (iii) specific geographical location, age and season for GI parasite egg/oocyst counts; and (iv) sex, season, specific geographical location, *Brucella* serology and lungworm and GI parasite egg/oocyst counts for metatarsus marrow fat, and subsequently the same variables and the marrow fat for the hunter condition assessment. For GI parasite egg/oocyst and lungworm larval counts, all animals with an imputed value of ≤0.1 epg or lpg (the lowest amount of eggs/oocysts or L1 measured above zero) were considered not infected and assigned a count of zero. We assumed that the response variable, qiviut cortisol, followed a gamma distribution as its values were positive and right-skewed, and *Up_lpg* was rescaled using a square-root (sqrt) transformation for better model fit.

Parallel Markov chain Monte Carlo (MCMC) chains were run with 900 000 iterations per chain, a burn-in period of 10 000 iterations and thinning of 1000. Results reported here were based on two chains, whereas two additional chains were run (four chains in total) for model diagnostics. Marginal posterior distributions and the parameter trace plots were examined to check the convergence of the chains. Mixing of the chains was assessed visually using the MCMC trace plots for each parameter (Supplementary Fig. 4) and the Gelman–Rubin diagnostic, with the potential scale reduction factor and effective sample size adjusted for autocorrelation, can be found in Supplementary Table 3. Posterior median estimates and 95% CrIs are shown in the Results.

All statistical analyses were performed using R version 3.6.1, and *rjags* version 4-10 and *coda* version 0.19-4 packages were used for Bayesian data analysis and diagnostics (Plummer *et al.,* 2006; Plummer, 2019; R Core Team, 2019).

## Results

When assessing the two-by-two correlations between variables, we found a fairly high correlation only between *Up_lpg* and *Ve_lpg* (i.e. r_s_ = 0.69, *P* < 0.001; all other r_s_ were <0.40). However, we included both *Up_lpg* and *Ve_lpg* in the model selection process for research purposes as the two lungworms differ in their life history characteristics (e.g. fecundity, life span), morphology and pathology (Kutz *et al.,* 1999; Verocai *et al.,* 2014; Kafle *et al.,* 2017). The final model obtained after manual backward elimination included *sex*, *season*, *year*, *location*, *marrow_fat*, sqrt(*Up_lpg*), as well as the interactions between *location* and sqrt(*Up_lpg*) and between *sex* and *season*.

**Figure 3:**
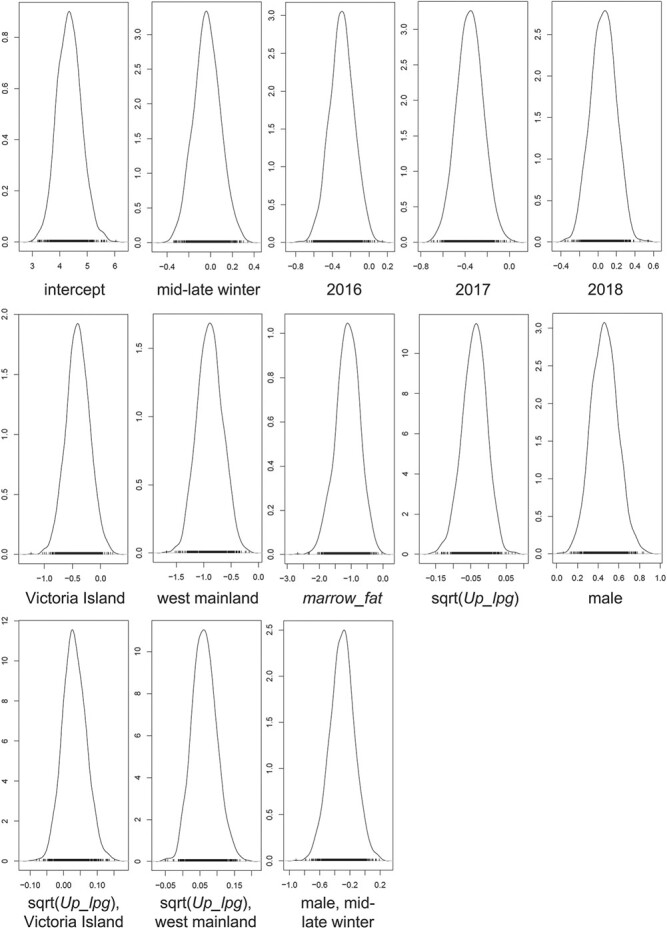
Marginal posterior distribution of the parameters. Y-axes correspond to the density, and X-axes correspond to the parameters. The peak of each distribution corresponds to the most likely parameter estimate; its spread corresponds to uncertainty about the parameter estimate. Positive values of a parameter imply that an increase in its variable is associated with higher qiviut cortisol levels, whereas negative values of a parameter imply that an increase in its variable is associated with lower qiviut cortisol levels. The intercept corresponds to the average qiviut cortisol values of females from the east mainland population in late fall–early winter 2015. All values shown are on the natural-logarithmic scale.

**Figure 4:**
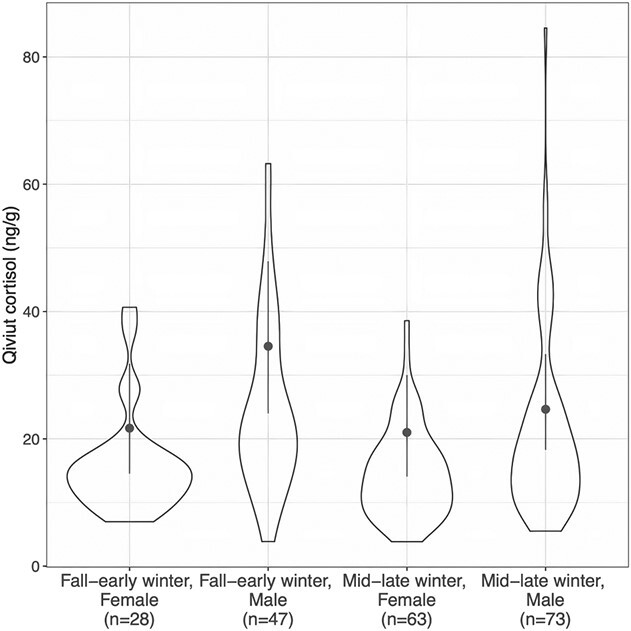
Relative qiviut cortisol levels of male and female muskoxen in fall–early winter and mid-winter–late winter. The violin plots represent the raw data. The dots show the posterior medians (the most likely estimates), and the lines show the 95% credible intervals (the uncertainty about the estimates) for qiviut cortisol by *sex* and *season*. All other categorical variables were fixed at the reference group and continuous variables at the median.

**Figure 5:**
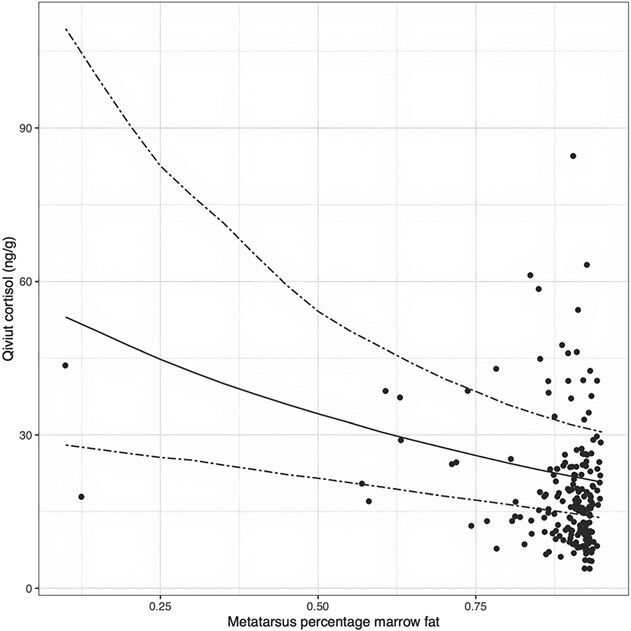
Effect of metatarsus percentage marrow fat on qiviut cortisol levels. The scatterplot shows the raw data. The solid line shows the posterior medians (the most likely estimates), and the dashed lines show the 95% credible intervals (the uncertainty about the estimates) for qiviut cortisol by metatarsus percentage marrow fat. All other categorical variables were fixed at the reference group and continuous variables at the median.

Overall, males had higher qiviut cortisol levels than females and qiviut cortisol levels did not differ between late fall–early winter and mid-winter–late winter when both sexes were considered together (Table 3; Fig. 3). There appeared to be an interaction between *sex* and *season*, with males having lower qiviut cortisol levels in mid-winter–late winter than in late fall–early winter, whereas females had similar qiviut cortisol levels during the two seasons (Fig. 4). Metatarsus marrow fat was negatively associated with qiviut cortisol (Fig. 5). Qiviut cortisol levels were lower in 2016 and 2017 than in 2015, but similar in 2015 and 2018, and decreased from the east mainland (Kent Peninsula) to Victoria Island (Lady Franklin Point, Ekaluktutiak and Ulukhaktok) to the west mainland (Kugluktuk) (Table 3; Fig. 3).

**Table 3:** Final model parameter posterior estimates (median and 95% CrI). The reference group for each categorical variable is listed first and all coefficients are on the natural-logarithmic scale.

Variable	Levels	Median	95%CrI
intercept		4.337	(3.534, 5.214)
*season*	Late fall–early winter Mid-winter–late winter	–0.034	- (−0.264, 0.210)
*year*	2015	-	-
	2016	−0.298	(−0.557, −0.041)
	2017	−0.356	(−0.580, −0.123)
	2018	0.061	(−0.193, 0.310)
*location*	East mainland	-	-
	Victoria Island	−0.404	(−0.813, 0.003)
	West mainland	−0.890	(−1.313, −0.450)
*marrow_fat*		−1.098	(−1.901, −0.404)
sqrt(*Up_lpg*)		−0.038	(−0.108, 0.025)
*sex*	Female	-	-
	Male	0.460	(0.225, 0.716)
sqrt(*Up_lpg*) and *location* interaction	Victoria Island	0.031	(−0.034, 0.102)
	West mainland	0.062	(−0.002, 0.137)
*sex* and *season* interaction	Male, mid-winter–late winter	−0.297	(−0.628, 0.015)

**Figure 6:**
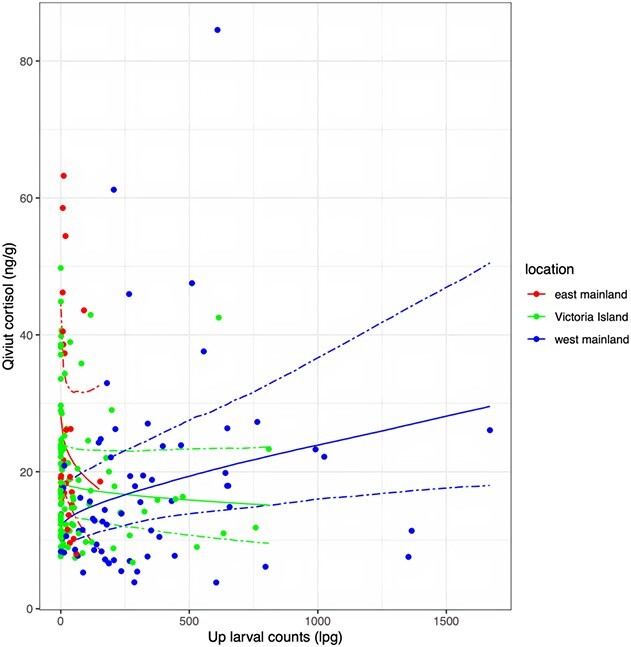
Effect of *U. pallikuukensis* (*Up*) larval counts on qiviut cortisol levels by *location*. The scatterplots show the raw data. The solid line shows the posterior medians (the most likely estimates), and the dashed lines show the 95% credible intervals (the uncertainty about the estimates) for qiviut cortisol by *location* and *Up* larval counts. All other categorical variables were fixed at the reference group and continuous variables at the median. The x-axis shows the posterior values up to the maximum value of *Up* larval counts within each location.

**Figure 7:**
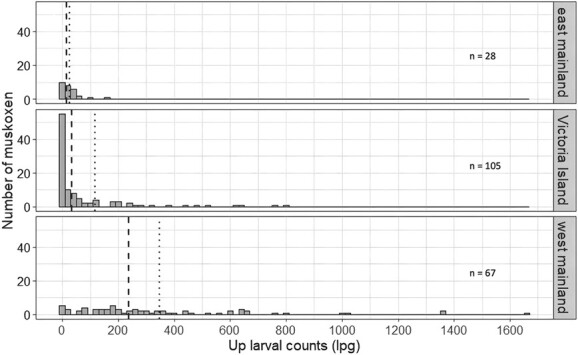
Histogram of *U. pallikuukensis* (*Up*) larval counts by *location*. Dashed lines and dotted lines indicate the median and mean infection intensities, respectively.

There appeared to be an interaction between *Up_lpg* and *location*. Qiviut cortisol levels tended to increase as *Up* larval counts increased on the west mainland, while they tended to decrease as *Up* larval counts increased on the east mainland, and no association was detected between qiviut cortisol and *Up* larval counts on Victoria Island (Table 3; Fig. 6). *Umingmakstrongylus pallikuukensis* abundance differed among these locations. Prevalence was 100% on the mainland (both east and west), whereas it was 72.4% on Victoria Island. Infection intensity also varied across sites: it was very low on Victoria Island [median (range) = 35.6 lpg (0.2–809.5); *n* = 76 (*n* = 29 were negative)] and the east mainland [median (range) = 16.6 lpg (0.2–153.2); *n* = 28], and much higher on the west mainland [median (range) = 236.5 lpg (0.4–1669.8); *n* = 67] (Fig. 7).

When added to the final model, back fat thickness (*n* = 145), incisor breakage score (*n* = 152) and their corresponding interactions had no effect on qiviut cortisol (results not shown).

## Discussion

We evaluated the associations between hair GCs and multiple measurable intrinsic and extrinsic factors. We identified several important influential factors (i.e. sex, season, qiviut growth year, and geographical location) and relationships between hair GCs and metrics of health (i.e. body condition and pathogen exposure/infection intensity metrics), as well as interactions between geographical location and *Up* larval counts, and between sex and season.

Qiviut cortisol levels were higher in males than in females, consistent with previous findings in wild muskoxen (Di Francesco *et al.,* 2017). Studies in a variety of wild mammalian species have differing results regarding variations in hair cortisol concentrations (HCCs) between sexes. Multiple studies report no differences in HCCs between sexes (Macbeth *et al.,* 2010; Malcolm *et al.,* 2013; Terwissen *et al.,* 2013; Yamanashi *et al.,* 2013; Carlitz *et al.,* 2014; Caslini *et al.,* 2016; Potratz *et al.,* 2019), whereas others document higher HCCs in males (Lafferty *et al.,* 2015; Schell *et al.,* 2017; Azevedo *et al.,* 2019; Santangeli *et al.,* 2019; Madslien *et al.,* 2020) or higher HCCs in females (Bechshøft *et al.*, 2011; Laudenslager *et al.,* 2012; Cattet *et al.,* 2014; Dettmer *et al.,* 2014; Dulude-de Broin *et al.,* 2019). Variations in HCCs between sexes may reflect sex differences in basal HPA-axis activity or response to stressors (Rhodes and Rubin, 1999; Levine, 2002; Turner *et al.,* 2002), as well as the experience of sex-specific stressors (e.g. rutting activity versus lactation) (Di Francesco *et al.,* 2017; Di Francesco *et al*., in press). Sex differences in HCCs could also be an artefact of hair growth patterns and fibre morphological characteristics, which may influence cortisol incorporation into hair (Dulude-de Broin *et al.,* 2019). Male muskoxen have coarser qiviut fibres and a higher variability in fibre diameter than females (Rowell *et al.,* 2001) and timing of qiviut shedding as well as onset of qiviut growth differ between sexes (Wilkinson, 1974; Gray, 1987; Robertson, 2000). Increased faecal GC metabolite (FGM) levels in males during the rut are documented for several ungulate species (Mooring *et al.,* 2006; McCoy and Ditchkoff, 2012; Corlatti *et al.,* 2014). Additionally, according to Indigenous knowledge holders, male muskoxen experience high stress during this period (Di Francesco *et al*., in press). The rut is characterized by reduced nutritional intake and repeated and prolonged aggressive interactions with male counterparts, along with courtship behaviours, which are all highly energetically demanding (Di Francesco *et al*., in press; Gray, 1987). The lower qiviut cortisol levels of males measured in mid-winter–late winter may reflect that highly stressed individuals are less likely to survive post-rut (which occurs from August to October; see Di Francesco *et al*., in press), but may also be linked to the lower stress experienced after the rut, which may dilute the qiviut cortisol levels, although lower qiviut growth occurs after September. Female qiviut cortisol levels did not differ between the two seasons. Based on the qiviut growth cycle, approximately April–November, qiviut cortisol levels may have been affected by parturition, lactation and early pregnancy. These reproductive status data were not available, thus we were unable to assess their influence.

Although we did not detect any age differences, this may reflect the limitations of the unbalanced dataset with a low number of juveniles and the coarse scale age classification (i.e. only two categories; Table 1). In studies on white-tailed deer (*Odocoileus virginianus*) and red deer (*Cervus elaphus*), HCCs did not vary significantly among fawns, yearlings and adults (>2 years) (Caslini *et al.,* 2016; Potratz *et al.,* 2019), whereas age differences were detected in moose (*Alces alces*) and Rocky Mountain goats (*Oreamnos americanus*) (Dulude-de Broin *et al.,* 2019; Madslien *et al.,* 2020). Such differences among age classes have been attributed to variations in HPA axis activity and/or in hair growth and moulting patterns (Azevedo *et al.,* 2019; Dulude-de Broin *et al.,* 2019).

Qiviut cortisol levels varied among geographical locations. They were lowest in the stable muskox population from the west mainland (Kugluktuk, NU MX-09 and west section of NU MX-11), higher in the declining Victoria Island population and highest on the east mainland (Kent Peninsula, east section of NU MX-11, population status unknown) (Leclerc, 2014; Tomaselli *et al.*, 2018b; Mavrot *et al*., unpubl. data). Differences in qiviut cortisol among geographical locations may reflect variations in biotic and abiotic environmental characteristics. Kugluktuk area is located in a different Bioclimate Subzone from Kent Peninsula and Victoria Island (Walker *et al.,* 2005). Additionally, Kugluktuk IK holders described that Victoria Island has a dryer land, poorer vegetation (both in quantity and quality) and a greater relative increase in predators compared to around Kugluktuk (Di Francesco *et al*., in press).

Qiviut cortisol varied among years. Inter-annual variations in HCCs were previously detected in muskoxen (different set of data) (Di Francesco *et al.,* 2017) and are described in other mammalian species, such as snowshoe hares (*Lepus americanus*) and rhesus monkeys (*Macaca mulatta*), in which higher HCCs were associated with increased predation risk and population density, respectively (Dettmer *et al.,* 2014; Lavergne *et al.,* 2020). Multiple biotic and abiotic factors may contribute to inter-annual differences in qiviut cortisol levels, such as variations in density, intraspecific competition, predator abundance, weather conditions, exposure to pathogens, food availability and abundance, human disturbance, etc. (Di Francesco *et al.,* 2017; Cuyler *et al.*, 2020b; Di Francesco *et al*., in press). The impact of some of these factors on GC levels has previously been highlighted in other wildlife species [e.g. anthropogenic activities and disturbances (Ewacha *et al.,* 2017; Périquet *et al.,* 2017; Santos *et al.,* 2018), predation risk (Brunton *et al.,* 2020; Lavergne *et al.,* 2020), weather conditions and events (Cizauskas *et al.,* 2015; Fardi *et al.,* 2018)]. These factors could not be included in our analyses but are likely important drivers of physiological stress in muskoxen, which are crucial to understand in the context of accelerating climate change. However, our results indicate that yearly qiviut cortisol levels may serve as a useful tool to monitor the combined effects of these factors on HPA axis activity at a population level.

Qiviut cortisol was inversely associated with marrow fat percentage but not with the other two body condition metrics—hunter assessment and back fat thickness. In ungulates, as an animal’s nutritional status declines, fat stores are generally used sequentially, starting with subcutaneous deposits (back fat), followed by visceral deposits and ultimately by bone marrow fat (Harris, 1945; Bear, 1971). The presence of an association between qiviut cortisol and *marrow_fat*, but not with *back_fat* or *condition_hunter* may attest to some resilience that muskoxen have up to a critical threshold, at which point the marrow fat starts declining and we begin observing an association with qiviut cortisol levels. The course scale for *condition_hunter* may not have allowed differentiation of animal condition once visible body fat stores were depleted. Alternatively, the sample sizes for these two alternate measures may have been too small to detect an effect in the final model. While the cause and effect relationship remains unclear, multiple studies in wild mammalian species have reported negative associations between body condition metrics and HCCs (Macbeth *et al.,* 2012; Cattet *et al.,* 2014; Mislan *et al.*, 2016; Potratz *et al.,* 2019; Shave *et al.,* 2019) or FGM levels (Cabezas *et al.,* 2007; Pokharel *et al.,* 2017; Wolf *et al.,* 2018). Elevated levels of circulating GCs may cause a reduction in body condition through the mobilization of energy and the inhibition of protein synthesis (Landys *et al.,* 2006; Cabezas *et al.,* 2007; Sapolsky *et al.,* 2000). Alternatively, nutritional stress may lead to reduced body condition and increased levels of circulating GCs as animals must mobilize energy stores (e.g. Jeanniard du Dot *et al.,* 2009; Bryan *et al.,* 2013). The combination of high qiviut cortisol and poor body condition in a muskox may, therefore, reflect not only nutritional stress, but also other long-term stressors (Cattet *et al.,* 2014; Mislan *et al.*, 2016).

The relationship between GC secretion and pathogen infections is highly complex and likely bidirectional, influenced by a multitude of factors related to the host and pathogen (e.g. pathogen type, host sex and age) (reviewed in Defolie *et al.,* 2020). In this study, we only detected associations between *Up* larval counts and qiviut cortisol. The cross-sectional nature of our study (i.e. observations of individuals occurred at a single time point; Carlsson *et al.,* 2019), the unique life histories of different pathogens and time lags between infection, cortisol incorporation into qiviut and sampling complicate interpretation of results.

The associations between *Up* larval counts and qiviut cortisol levels varied among locations. Consideration for the dynamic recent history of geographic colonization of this parasite provides some insights into these enigmatic findings. *Umingmakstrongylus pallikuukensis* is well established and abundant near Kugluktuk and has recently expanded its geographic range northward and eastward in response to rapid Arctic climate warming (Kafle *et al.,* 2020). It was first detected on Victoria Island in 2008 near Lady Franklin Point and near Ekaluktutiak in 2012 (Kutz *et al.*, 2013a); recent range expansion east of Kugluktuk towards Kent Peninsula has also recently occurred, but the parasite remains at lower prevalence and intensity in this area. The positive relationship with qiviut cortisol in Kugluktuk suggests a measurable impact associated with the high intensities of infection with *Up* in muskoxen in this region. In contrast, absence of a relationship on Victoria Island suggests that the parasite cost may be minimal at low intensities. The apparent inverse association on Kent Peninsula remains enigmatic but, given the small sample size (*n* = 30) and very low intensities of infection, it is perhaps a spurious relationship. We did not observe a relationship between qiviut cortisol levels and *Ve* larval counts or lungworm richness. *Varestrongylus eleguneniensis* was present at a lower prevalence and infection intensity in all three locations and is substantially smaller than *Up* with presumably less of an impact on muskoxen (Verocai *et al.,* 2014). Two studies investigating the relationship between GC levels and protostrongylid lungworm infections in ungulates found conflicting results. A lungworm removal experiment in bighorn sheep (*Ovis canadensis canadensis*) showed no difference in FGM levels between the treated and control herds (Goldstein *et al.,* 2005), whereas another study found a positive correlation between lungworm larval output and FGM levels in male Alpine chamois (*Rupicapra rupicapra*) (Hoby *et al.,* 2006).

We did not find a relationship between GI parasite burdens and qiviut cortisol levels. This is consistent with other studies on naturally infected Artiodactyls measuring HCCs (e.g. Trevisan *et al.,* 2017) or FGM levels (e.g. Cizauskas *et al.,* 2015). The absence of association between qiviut cortisol and GI parasites in our study may be linked to the fairly low infection intensities, but it may also suggest that muskoxen use a tolerance strategy to deal with GI parasites. This has previously been suggested by Carlsson *et al.* (2016a) for reindeer (*Rangifer tarandus tarandus*), and Cizauskas *et al.* (2015) for plains zebras (*Equus quagga*) and springboks (*Antidorcas marsupialis*). Tolerance mechanisms generally evolve when virulence is low and rate of transmission is high (Restif and Koella, 2004).

Our opportunistic sampling design was a limiting factor for assessing the relationship between qiviut cortisol and parasite burden. Many Arctic parasites are highly seasonal (Kutz *et al.,* 2012) and this can result in a temporal mismatch between the parasite burden measured through faecal larval/egg/oocyst counts at time of sampling (late fall/winter) and the parasite burden present during the summer qiviut growth period. This mismatch requires further exploration of the specific life cycle and thermal tolerances of each parasite species. For example, *T. boreoarcticus*, the dominant GI parasite of muskoxen, is most abundant and pathogenic from late spring to late summer, during peak qiviut growth. It produces eggs mainly throughout the summer and the few eggs produced during winter do not survive freezing, thus the association between this parasite and qiviut cortisol could not be evaluated based on our late fall/winter sampling design (Kutz *et al.,* 2012). *Marshallagia marshalli*, another common abomasal nematode parasite of muskoxen, is transmitted throughout the year with peak faecal egg counts in winter. Mid-winter–late winter burdens may reflect recent acquisition during a time when the qiviut was no longer growing, and thus effects of the parasite on the HPA axis would not likely be reflected in the qiviut. In contrast, *Up* and *Ve* both have long pre-patent (time from exposure to presence of reproductively active adult parasites) and patent periods (lifespan of the adult parasites) and winter infection intensities may be associated with summer stressors that resulted in increased exposure or increased susceptibility to infection (Kutz *et al.,* 1999; Kafle *et al.,* 2017). However, effects of the winter burdens of adult parasites would not be reflected in the qiviut cortisol. For the intestinal protozoan *Eimeria* spp., high oocyst shedding generally occurs in immediate response to recent stress (Pyziel *et al.,* 2011; Chartier and Paraud, 2012); this also would not be detectable in the qiviut in the absence of growth (Di Francesco *et al.,* 2021). A shorter-term measure of GC levels, such as faecal GC metabolites, would provide complementary information to better unravel the complex relationships between parasite burden and GC levels.

We did not detect an association between qiviut cortisol levels and *E. rhusiopathiae* or *Brucella* exposure status. Seropositivity indicates exposure to these two bacteria, but does not provide information regarding the timing of exposure, occurrence of clinical disease or the subsequent possible recovery of the animal. *Erysipelothrix rhusiopathiae* was associated with acute mortality in muskoxen of the western Canadian Arctic archipelago (Kutz *et al.,* 2015; Forde *et al.,* 2016). While there is evidence of very few carrier animals, no chronic forms have been described in this species (see Forde *et al.,* 2016). Assuming that positive cases of *E. rhusiopathiae* among our harvested animals are recovered, it is not surprising that we did not detect an association between exposure to this bacterium and qiviut cortisol levels. In contrast, *Brucella suis* biovar 4, reported increasingly in muskoxen from Victoria Island, is associated with chronic disease manifestations (Tomaselli *et al.*, 2018b, 2019). We would have predicted higher qiviut cortisol levels in *Brucella*-positive muskoxen as chronic illnesses have previously been associated with increased HCCs in domestic cattle (*Bos taurus*) (Braun *et al.,* 2017). The absence of association, however, may be due to the animals being exposed to the bacterium after the qiviut had stopped growing and/or to the unbalanced dataset and low prevalence.

The simultaneous infection of muskoxen by multiple pathogen species was common in this study; however, we only investigated associations between qiviut cortisol and single pathogens. Co-infecting pathogens may have synergistic or antagonistic interactions, which modify their impact on the infected host, when compared to single-pathogen infections (Joly and Messier, 2005; Jolles *et al.,* 2008; Thumbi *et al.,* 2013). Identifying and further exploring such interactions among pathogens and their association with qiviut cortisol may elucidate new insights.

We did not detect an association between qiviut cortisol and incisor breakage score. Severe wearing or breakage of incisors may affect browsing and food processing efficiency, leading to nutritional deficiencies (Kojola *et al.,* 1998; Ericsson and Wallin, 2001). However, this effect may have been lower during the summer and fall, when the qiviut is growing, as the vegetation is more abundant and easier to access (Di Francesco *et al*., in press).

It is important to note that we encountered substantial computational limitations in the number of models we could fit due to the large amount of time necessary to run each MCMC analysis. We consequently chose to use two different stepwise variable selection procedures to decide which variables should be included in the model (i.e. addition of each interaction independently to the model with all the main effects to select the interactions and then backward elimination to simplify the model). However, there are important limitations associated with stepwise methods (e.g. bias in parameter estimation, problem of multiple hypothesis testing and increased type I error rates, etc.), which may have led to the exclusion of variables that were actually associated with qiviut cortisol, while including other variables that did not truly have an effect (Whittingham *et al.,* 2006; Mundry and Nunn, 2009).

This study has highlighted some important limitations and challenges, including issues associated with temporal mismatch between qiviut cortisol and various health determinants (e.g. parasites), which can inform the experimental design of future studies in wildlife endocrinology. Although we found multiple associations between qiviut cortisol levels and extrinsic factors and metrics of health, due to the cross-sectional nature of this study, we were unable to infer causal relationships. Future research should focus on more specifically elucidating the relationship between a particular stressor, the increased cortisol levels it causes and the subsequent effect this has on health and fitness. This could be done through longitudinal studies, by monitoring and resampling marked individuals over several years (Carlsson *et al.,* 2019). Such a long-term study of Rocky Mountain goats has recently demonstrated a link between increased predation risk and breeding suppression through chronically elevated cortisol levels (Dulude-de Broin *et al.,* 2020).

While we mostly focused on evaluating associations between qiviut cortisol levels and measures of health at an individual level, our results also highlighted inter-annual variations and suggested differences in qiviut cortisol among geographical locations that are consistent with the local muskox population trends. Gathering long-term data from additional muskox populations for which population trends, and preferably annual productivity, are known would provide further insights as to whether these associations between population trajectory and qiviut cortisol levels hold true. If increased GC levels are shown to precede population declines, poor productivity or mortality events, these could be used as predictive biomarkers of population health and fitness and inform management and conservation actions pro-actively.

This study was made possible by the fruitful collaborations developed with the communities of Ekaluktutiak, Kugluktuk and Ulukhaktok and the strong participation of local hunters in sample collection. Hunter-based sampling programs can contribute substantially to ecological and wildlife health studies (Carlsson *et al.*, 2016b; Kutz and Tomaselli, 2019; Tomaselli, 2019; Peacock *et al.,* 2020). In particular, for this study, hunter-based sampling allowed for collection of a standardized set of samples, from a large number (*n* = 219) of animals, across a broad geographic area and over several years. The collection of these samples would not have been logistically, financially nor ethically feasible using conventional scientific approaches.

Finally, in this study, we only included broad extrinsic factors (i.e. year and geographic location) in the analyses, which did not allow us to identify the specific drivers of physiological stress in muskoxen. Muskoxen are likely impacted by multiple factors, many of which are predicted to increase with accelerating climate change (e.g. insect harassment, rain on snow, heat extremes, changing vegetation, air quality, etc.) (see Cuyler *et al.*, 2020b; Di Francesco *et al*., in press). The use of both scientific knowledge and Indigenous knowledge to develop indicators/metrics for the various potential stressors of muskoxen (e.g. Di Francesco *et al*., in press; Peacock *et al.,* 2020), such as indexes of insect harassment (some have already been developed for reindeer and caribou; Weladji *et al.,* 2003; Witter *et al.,* 2012) and quantitative and/or qualitative measures of plant abundance and quality, will help to inform and guide future conservation endocrinology studies.

## Funding

J.D.F. was supported by the Natural Sciences and Engineering Research Council of Canada (NSERC)-Collaborative Research and Training Experience (CREATE) Host–Parasite Interactions Training Program; the NSERC-CREATE Integrated Training Program in Infectious Diseases, Food Safety and Public Policy; the University of Calgary Faculty of Graduate Studies; and the Morris Animal Foundation [Fellowship Training Grant D18ZO-407]. S.K. was supported by grants from Polar Knowledge Canada [Grant NST-1718-0015], NSERC [Discovery Grant RGPIN/04171-2014 and Northern Supplement RGPNS/316244-2014], ArcticNet, Canada North Outfitting and the Shikar Foundation.

## Data Availability Statement

The data underlying this article will be shared on reasonable request to the corresponding author.

## Supplementary Material

supplementary_coab103
