# Supplementary material for: Qiviut cortisol is associated with metrics of health and other intrinsic and extrinsic factors in wild muskoxen (Ovibos moschatus)
Source: Conserv Physiol. 2022 Jan 21;10(1):coab103. doi: 10.1093/conphys/coab103 (PMC9040286; doi:10.1093/conphys/coab103)
Supplement: supplementary_coab103 [file supplementary_coab103.zip › Sup_Fig1.pdf]

Supplementary Figure 1: Information form provided to the hunters with the kits.

MUSKOX SAMPLE COLLECTION

ANIMAL ID ☐

COMMUNITY : ☐

HUNTER'S NAME ☐ GUIDE'S NAME: ☐

CONTACT: ☐

KILL DATE : ___ / ___ / ___ (dd mm yy) TAG NUMBER: ☐

TYPE OF HUNT ☐ Community ☐ Individual ☐ Sport

KILL LOCATION : ☐ (eg. Nunatunek) MANAGEMENT ZONE: ☐

LATITUDE N ☐ LONGITUDE : W ☐

SEX : ☐ Female ☐ MalE ☐ Unknown

If female specify if: ☐ pregnant ☐ lactating ☐ none ☐ unknown

AGE CLASS : ☐ Calf ☐ Yearling ☐ 2-3 y old ☐ 3-4 y old ☐ >4 y old ☐ Unknown

BODY CONDITION : ☐ Really fat ☐ Fat ☐ Not bad ☐ Skinny

BACK FAT (measure with paper ruler on the provided leg tag) : _______________ cm

HERD SIZE N° : ☐ (all animals) N° CALVES : ☐ N° YEARLINGS : ☐

SAMPLES COLLECTION (check the box when collected):

☐ BLOOD ☐ FUR ☐ FECES ☐ LEFT HIND LEG ☐ BACK FAT ☐ JAW

☐ ABNORMAL TISSUE specify: ______________________________

NOTES: ______________________________
____
____
