# Supplementary material for: Qiviut cortisol is associated with metrics of health and other intrinsic and extrinsic factors in wild muskoxen (Ovibos moschatus)
Source: Conserv Physiol. 2022 Jan 21;10(1):coab103. doi: 10.1093/conphys/coab103 (PMC9040286; doi:10.1093/conphys/coab103)

Supplementary Figure 2: Percentage of missing values for each variable (a) and missing data by animal (b).

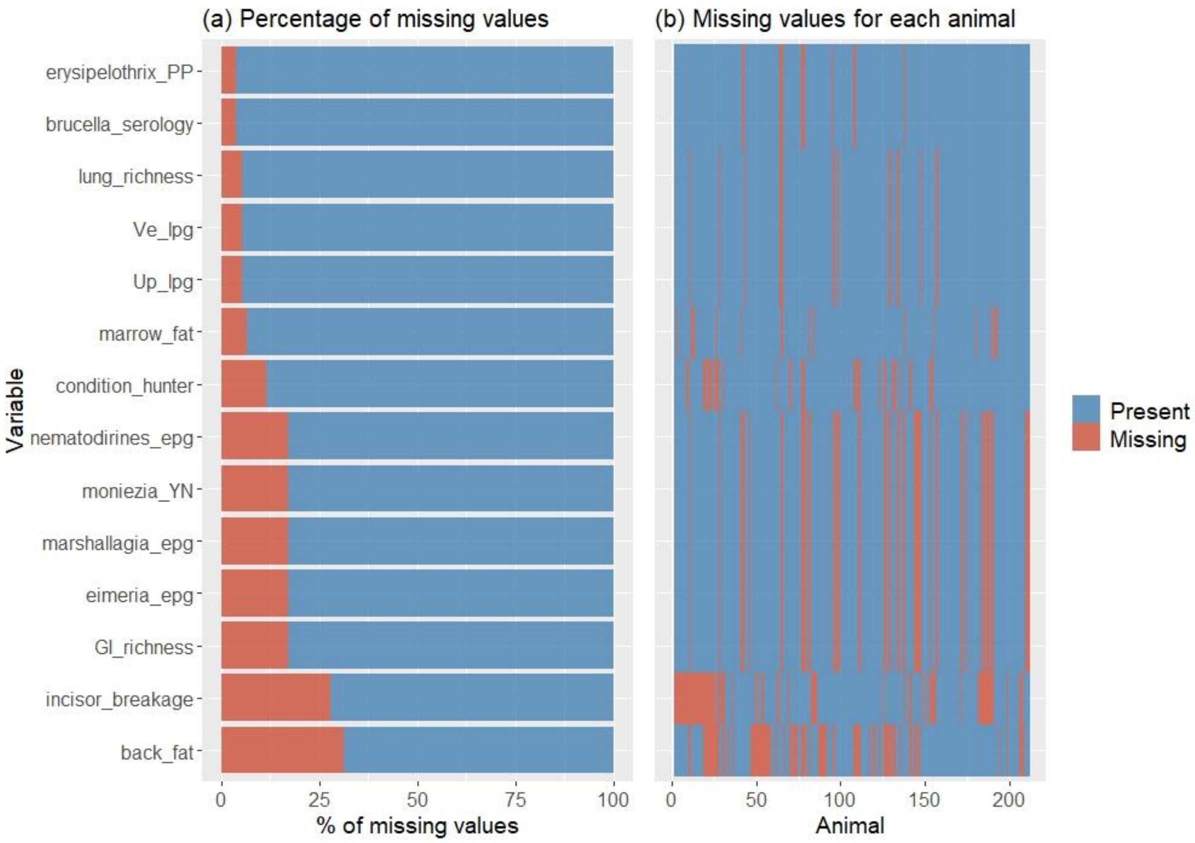

Supplement: supplementary_coab103 [file supplementary_coab103.zip › Sup_Fig2.pdf]
