# Supplementary material for: Qiviut cortisol is associated with metrics of health and other intrinsic and extrinsic factors in wild muskoxen (Ovibos moschatus)
Source: Conserv Physiol. 2022 Jan 21;10(1):coab103. doi: 10.1093/conphys/coab103 (PMC9040286; doi:10.1093/conphys/coab103)

Supplementary Figure 3: Boxplot of metatarsus length by age class for animals of known age class harvested on Victoria Island (n = 62 adults and n = 12 juveniles). The red line indicates the cut-off of 16 cm, above which muskoxen were classified as adults and below which the age remained "unknown."

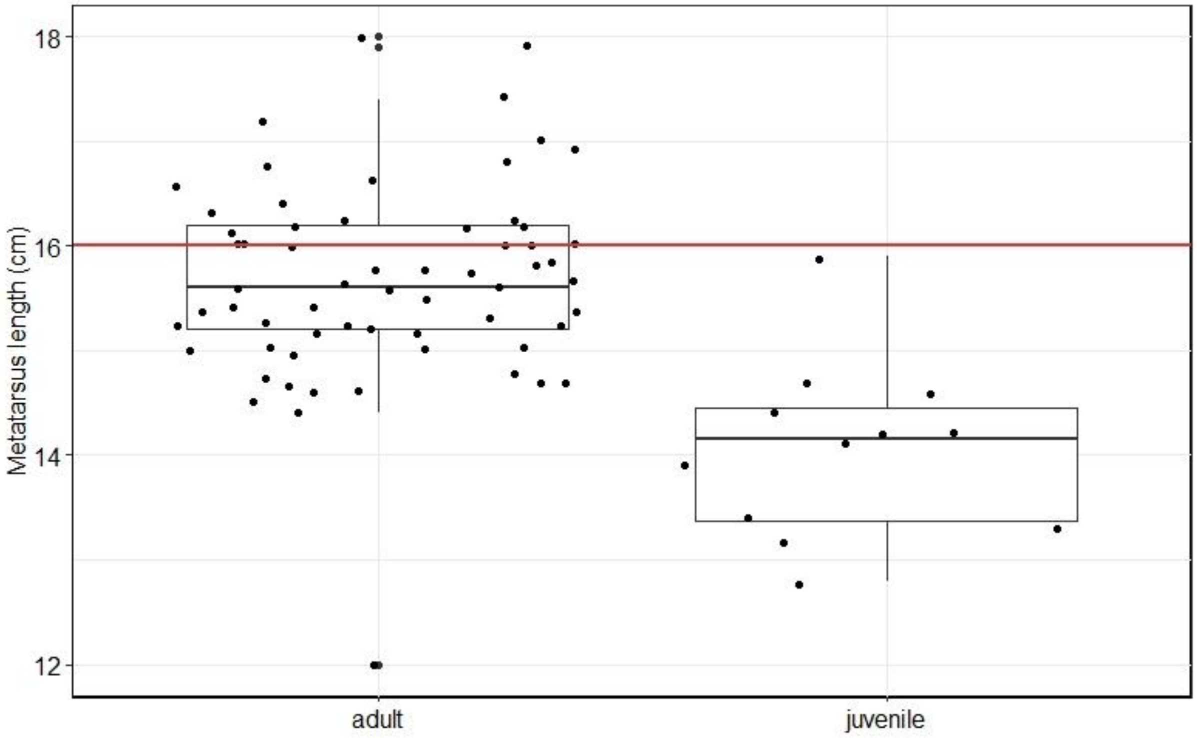

Supplement: supplementary_coab103 [file supplementary_coab103.zip › Sup_Fig3.pdf]
