# Supplementary material for: Qiviut cortisol is associated with metrics of health and other intrinsic and extrinsic factors in wild muskoxen (Ovibos moschatus)
Source: Conserv Physiol. 2022 Jan 21;10(1):coab103. doi: 10.1093/conphys/coab103 (PMC9040286; doi:10.1093/conphys/coab103)

Supplementary Figure 4: Parameter trace plots. Red, black, green, and blue represent the four Markov chain Monte Carlo chains. Plots show adequate mixing and convergence of the four chains.

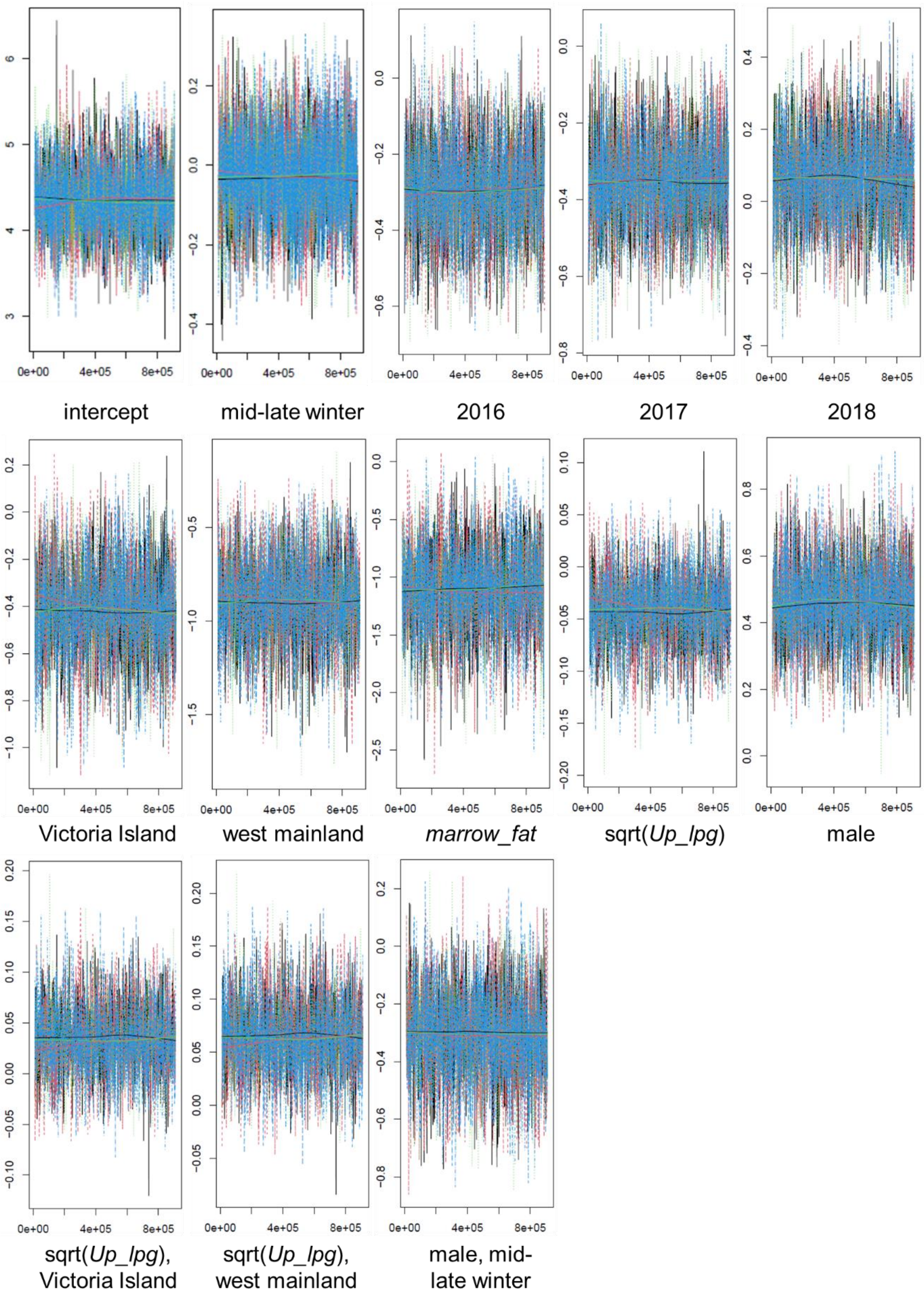

Supplement: supplementary_coab103 [file supplementary_coab103.zip › Sup_Fig4.pdf]
