# Supplementary material for: Qiviut cortisol is associated with metrics of health and other intrinsic and extrinsic factors in wild muskoxen (Ovibos moschatus)
Source: Conserv Physiol. 2022 Jan 21;10(1):coab103. doi: 10.1093/conphys/coab103 (PMC9040286; doi:10.1093/conphys/coab103)
Supplement: supplementary_coab103 [file supplementary_coab103.zip › Sup_Table1.pdf]

Supplementary Table 1: Investigation of biologically and ecologically plausible two-way interactions. Interactions were added individually to the model including all main effects (*sex, age, year, location, Up_lpg, Ve_lpg, lung_richness, nematodirus_epg, eimeria_epg, marshallagia_epg, moniezia_YN, GI_richness, condition_hunter, marrow_fat, erysipelothrix_PP*, *brucella_serology*) and their impact on the deviance information criterion (DIC) and potential effect were assessed. Interactions that gave the best improvement (i.e., highest decrease) of the DIC and those which had a potential effect on qiviut cortisol were retained for model building and are indicated in bold.

**Interaction**	**DIC**	**Potential effect**
None (i.e., model with all fixed effects)	6630.17	-
season:condition_hunter	6638.72	no
sex:erysipelothrix_PP	6638.47	no
year:brucella_serology	6636.61	no
age:monizeia_YN	6635.83	no
sex:age	6635.61	no
age:marrow_fat	6634.91	no
sex:condition_hunter	6634.57	no
age:season	6633.99	no
age:brucella_serology	6633.99	no
age:erysipelothrix_PP	6633.54	no
age:nematodirines_epg	6633.36	no
age:marshallagia_epg	6633.08	no
age:condition_hunter	6632.07	no
location:erysipelothrix_PP	6631.71	no
age:eimeria_epg	6630.98	no
age:Up_lpg	6628.90	no
location:Up_lpg	**6628.66**	**yes**
year:season	6626.94	no
season:marrow_fat	-	- (coefficients not converged)
sex:season	**6624.81**	**yes**
sex:brucella_serology	6624.53	no
sex:marrow_fat	6622.71	no
location:Ve_lpg	6622.10	no
age:Ve_lpg	**6620.41**	**no**
sex:year	**6618.06**	**yes**
year:erysipelothrix_PP	**6616.51**	**no**
