# Supplementary material for: Qiviut cortisol is associated with metrics of health and other intrinsic and extrinsic factors in wild muskoxen (Ovibos moschatus)
Source: Conserv Physiol. 2022 Jan 21;10(1):coab103. doi: 10.1093/conphys/coab103 (PMC9040286; doi:10.1093/conphys/coab103)
Supplement: supplementary_coab103 [file supplementary_coab103.zip › Sup_Table2.pdf]

Supplementary Table 2: Model selection procedure by manual backward stepwise elimination.

	**DIC**
**"Stage 2" starting model** (*sex, age, year, location, Up_lpg, Ve_lpg, lung_richness, nematodirus_epg, eimeria_epg, marshallagia_epg, moniezia_YN, GI_richness, condition_hunter, marrow_fat, erysipelothrix_PP, age:Ve_lpg, sex:year, year:erysipelothrix_PP, location:Up, sex:season*)	6634.36
Removal of *GI_richness*	6630.78
Removal of GI parasites egg/oocyst counts (*nematodirines_epg, eimeria_epg, marshallagia_epg, moniezia_YN*)	6616.10
Removal of *condition_hunter*	6204.61
Removal of *lung_richness*	6198.32
Removal of *year:erysipelothrix_PP*	6188.88
Removal of *erysipelothrix_PP*	6048.36
Removal of *sex:year*	6038.72
Removal of *age:Ve_lpg*	6039.56
Removal of *Ve_lpg*	6036.66
Removal of *age* (**final model with *sex, year, location, Up_lpg, marrow_fat, location:Up, sex:season***)	**6033.43**
