# Supplementary material for: Qiviut cortisol is associated with metrics of health and other intrinsic and extrinsic factors in wild muskoxen (Ovibos moschatus)
Source: Conserv Physiol. 2022 Jan 21;10(1):coab103. doi: 10.1093/conphys/coab103 (PMC9040286; doi:10.1093/conphys/coab103)
Supplement: supplementary_coab103 [file supplementary_coab103.zip › Sup_Table3.pdf]

Supplementary Table 3: Gelman–Rubin diagnostic evaluating the convergence between the four Markov chain Monte Carlo chains – Potential scale reduction factor and effective sample size.

**Parameters**	**Point estimate**	**Upper Confidence Interval**	**Effective sample size**
intercept	1.000	1.002	3334.745
Up_lpg	1.002	1.007	2928.954
season, mid-late winter	1.000	1.002	3745.742
year, 2016	1.000	1.000	3833.518
year, 2017	1.000	1.000	3778.619
year, 2018	1.000	1.000	3528.927
location, Victoria Island	1.000	1.002	2891.048
location, west mainland	1.000	1.001	3168.620
location.Up_lpg, Victoria Island	1.002	1.006	3033.741
location.Up_lpg, west mainland	1.002	1.006	2956.153
marrow_fat	1.001	1.003	3404.592
sex, male	1.001	1.002	3967.086
sex.season, male, mid-late winter	1.001	1.002	3709.510
